# Carbon Dioxid in the Primary Cultivation of the Gonococcus

**Published:** 1919

**Authors:** 


					﻿Carbon Dioxid in the Primary Cultivation of the Gonococcus.
By Charles W. Chapin. From the Laboratory of the U. S.
Immigration Station, Ellis Island, N., Y., and the Labora-
tory of the U. S. Marine Hospital, Stapleton, N. Y.
Journal of Infectious Diseases, October, 1918.
The author reports results of investigation of the cultural require- '
ments of the gonococcus, with carbon dioxid at the same pressure
as that in living tissue, viz : 5-7 cm. Hg.
A meat infusion was prepared in the usual way, and to it were
added urine 20 0/0, peptone 0.5 0/0, and a normal dilution of sul-
phuric acid 1.2 0/0. The mixture was divided in five flasks and
different percentages of normal solution sodium hydroxid added.
The reaction readings were then taken. The finished medium also
contained agar, q. s., pea flour 0.5 0/0, dextrose 0.5 0/0 and egg-yolk
5 0/0, the yolk being added to the cooled medium just before pour-
ing the plates. Four plates were poured from each flask, and on
the surface of the plates was spread the slightly diluted gonorrhoeal
pus. Glass covers were put over them and they were placed in
iwo stacks in a pan, covered with mouse-jars, and sealed with a
’little water. The carbon dioxid was treated with sodium bicarbon-
ate and sulphuric acid and collected over water to which a little
sodium bicarbonate had been added. To the atmosphere of a jar
the carbon dioxid (approximately 10 0/0) was added, and the unal-
tered atmosphere of the other jar served as control.
A wide range of reactions was shown. Carbon dioxid might bo-
expected to make more favorable an alkaline medium, but it
appears to have improved the conditions even with highly acid
media.
TABLE
Normal NaOH per cent.	o	1.2	2.3	3.7	4.7
Reaction............ 3.4	2.6	1.8	0.5	alk.
Control........................... 1	1
1	2
Carbon Dioxid ....	3	10	10	10	10
3	10	10	8	10
Experiments give distinct evidence that carbon dioxid improves
acid media by some effect on the raw proteid, but data is here
insufficient.
Wherry and Oliver show that the gonococcus grows best at
oxygen pressure below that of air. A lighted candle placed in the
jar at time of sealing establishes a favorable atmosphere.
Author’s summary : “ An atmosphere rich in carbon dioxid has
been found to facilitate the primary cultivation of the gonococcus
on mediums containing raw proteid ”.
				

